# Impact of operation duration on short-term and long-term prognosis in patients undergoing radical colorectal surgery

**DOI:** 10.7150/jca.65817

**Published:** 2022-01-16

**Authors:** Xuefang Shen, Changming Zhou, Qing Hua, Liu Yang, Weiwei Zhao, Pingbo Xu

**Affiliations:** 1 Department of Anesthesiology, Fudan University Shanghai Cancer Center, Shanghai, China.; 2 Department of Cancer Prevention, Fudan University Shanghai Cancer Center, Shanghai, China.; 3 Department of Integrated Therapy, Fudan University Shanghai Cancer Center, Shanghai, China.; 4 Department of Oncology, Shanghai Medical College, Fudan University, Shanghai, China.

**Keywords:** operation duration, short-term and long-term prognosis, colorectal cancer, radical surgery

## Abstract

**Purpose**: The objective of this study was to evaluate the impact of operation duration on short- and long-term outcomes of colorectal cancer patients following surgical resection.

**Methods**: 6224 consecutive patients who underwent radical colorectal surgery were retrospectively assessed and were divided into short operation duration group (SOD) and long operation duration group (LOD) according to the operation duration cutoff value of 110 minutes.

**Results**: Compared with patients in LOD group, patients in SOD group had significantly lower total costs in hospital, reduced expenses for drugs and antibiotics, shorter length of stay (LOS) in hospital and in the ICU. Moreover, 5-year overall survival (OS) and disease-free survival (DFS) for patients in the SOD group were markedly higher than for patients in the LOD group. Mutivariate regression analysis indicated that longer operation duration was associated with poor prognosis, with a hazard ratio of 1.004 (1.003, 1.005) for OS and 1.005 (1.003, 1.006) for DFS. Finally, surgeons' qualifications had meaningful correlation with operation duration (r= 0.450).

**Conclusions**: Operation duration is an independent risk factor for patients' short-term and long-term outcome after radical colorectal surgery. Improve the surgical skills of the surgeon may shorten the operation duration, and further improve the outcome for patients.

## Introduction

According to global cancer statistics for 2018, colorectal cancer (CRC) was the fourth most commonly diagnosed cancer and the second highest cause of cancer-related death 1. Radical surgical resection is currently the most common approach for colorectal cancer treatment 2. Optimizing surgical processes may affect postoperative prognosis, which is consistent with the notion of enhanced recovery after surgery (ERAS).

Patient characteristics, such as pathologic Tumor-Node-Metastasis (pTNM), poor differentiated clusters of neoplastic cells 3, and tumor location 4 are important factors that affect outcomes. However, operation duration is one of the key operational factors. Growing bodies of studies suggest that long operation duration is associated with poor perioperative prognosis, such as venous thromboembolism (VTE) 5, infection 6, and even overall rate of perioperative complications 7.

Although there have been many studies concerning the association between operation duration and postoperative complications, there have been no consistent conclusions 6, 8-12. Some researchers have suggested that prolonged operation duration, especially lasting over 4 hours, can increase postoperative complications and length of stay (LOS) in hospital, and can have a negative impact on the outcome of enhanced recovery after laparoscopic colorectal resection 6, 8, 10, 11. Other studies have suggested that prolonged operation duration does not increase postoperative morbidity 9, 12. Moreover, only a few studies have focused on the effect of operation duration on long-term prognosis. Wang et al. found that prolonged operation duration was associated with periprosthetic joint infection within 1 year after primary joint arthroplasty 13. Additionally, there are currently few studies that have reported on the relationship between long-term prognosis and prolonged operation time in patients undergoing tumor resection. Therefore, the current study aimed to explore the effects of operation duration on postoperative short-term and long-term outcomes in a large cohort of patients undergoing radical resection for CRC.

## Materials and Methods

### Data collection

Between July 1, 2011 and December 31, 2016, all consecutive patients who underwent radical resection for colorectal cancer at Fudan University Shanghai Cancer Center (FUSCC) were recruited for this retrospective study. The inclusion criteria were as follows: (1) age >18 years; (2) standard open or laparoscopic radical resection of colorectal cancer confirmed by pathology; (3) local lesions without distant metastasis; (4) availability of all clinical data. Patients with metastasis, recurrence, or other palliative surgery were excluded. In addition, patients who underwent operations by surgeons with less than 10 years' surgical experience were excluded to guarantee the quality of the operation. From the database of the FUSCC clinical information system, medical information, demographic information, tumor location, pathological diagnosis, pathological stage, operation methods, extramural vascular invasion (EMVI), perineural invasion (PNI), circumferential radial margin (CRM), anesthesia methods, ASA class, routine blood tests, medical costs, and number of lymph nodes examined were extracted. The primary endpoint was the long-term prognosis for patients, including overall survival (OS) and disease-free survival (DFS). The secondary endpoint was the short-term prognosis, including costs, LOS in hospital, and LOS in the ICU. The last follow-up date was November 30, 2019. The study was approved by the institutional review committee of FUSCC (Shanghai, China). Informed consent was waived due to the retrospective nature of the study.

### Statistical methods

Mean ± standard deviation (SD) and median (q1, q3) were used to describe continuous variables, while frequencies and percentages were used for categorical variables. Chi-square test or Fisher's exact test (as appropriate) were applied when testing the variables' differences between different operation duration groups for categorical variables; Wilcoxon sum rank test or Kruskal-Wallis H test were applied when testing differences between different operation duration groups for continuous variables. The Kaplan-Meier method was used to calculate survival rate, and log-rank test was used to compare differences between the groups. Cox proportional hazards models were used to calculate the univariate and multivariate adjusted risk ratios (HRs) and 95% confidence intervals (CIs). Univariate and multivariate analyses were performed for all significant variables, except operation time, to identify survival-confounding factors. Then, propensity score matching (PSM) was utilized between groups to reduce the influence of these confounding factors. PSM was conducted in R (R Core Team, 2014) and the rest of the statistical analyses were performed using IBM SPSS Statistics for Windows, version 25.0 (IBM Corp., Armonk, N.Y., USA).

## Results

### Patient characteristics

Between 2011 and 2016, 9135 patients aged >18 years underwent colorectal resection, and their records were retrieved from the database. 879 patients with metastasis, recurrence, or palliative surgery, were excluded from them. Then, 2032 patients undergoing surgery by surgeons with less than 10 years' surgical experience were excluded to eliminate the influence of surgeons' experience on prognosis. Finally, 6224 patients with colorectal cancer were eligible for analysis. The patient attrition flow chart is shown in Supplementary Fig. 1.

The histogram of CRC patients' operative duration is shown in Supplementary Fig. 2. The levels of operative duration had positively skewed distributions. The mean (± SD) and median (q2, q3) of operation duration were 111.00 ± 41.35 and 102.00 (82.00, 131.00) minutes, respectively, with a minimum of 45 minutes and a maximum of 445 minutes. In order to facilitate the analysis of the relationship between operative duration and clinical outcomes, 110 minutes of operative duration was selected as the grouping criterion. Patients were then assigned into the SOD group (≤ 110 min, n = 3668) or LOD group (> 110 min, n = 2556).

The clinico-pathological characteristics of the study population according to the two groups are given in Table 1. Preoperative hemoglobin, platelet, serum creatinine and albumin levels, pTNM staging, EMVI+, PNI+, and number of lymph nodes examined were similar between the groups (*P* > 0.05). However, there was a greater proportion of female patients, older adults (≥ 65 years), overweight and obesity patients, laparoscopic surgery, carcinoma of the rectum (vs right or left colon cancer), signet-ring cell carcinoma (vs adenocarcinoma or mucinous adenocarcinoma), CRM+, combined anesthesia (GA+EA), and ASA III-V (vs I or II) in the LOD group compared with the SOD group (*P*<0.05). Then, the CRC patients in the SOD group and LOD group were separately matched (1:1) by PSM. The confounding factors, such as gender, age, surgical approach, pTNM staging, tumor site, histological type, EMVI, PNI, CRM, number of lymph nodes examined, hemoglobin, and albumin were well matched in the two cohorts, and none was found to differ significantly between the pairs at baseline (Supplementary Table 1 and Supplementary Fig. 3).

### Longer operation duration was related to poor prognosis for CRC patients

As shown in Table 1, patients in the SOD group vs the LOD group had lower total costs in hospital (43558 ± 13298 CNY *vs* 48768 ± 17031 CNY, respectively; *P*<0.001), lower drug costs (15529 ± 8129 CNY *vs* 17269 ± 9778 CNY; *P*<0.001) and lower antibiotic costs (939±1158 CNY *vs.* 1080 ± 1377 CNY, *P*<0.001), shorter LOS in hospital (14.91 ± 6.64 days vs 16.40 ± 7.41 days; *P*<0.001) and shorter LOS in the ICU (0.18 ± 1.17 days *vs* 0.24 ± 1.22 days; *P*=0.047). On univariate COX regression analysis, prolonged operation duration was found to be associated with higher total costs in hospital (especially the cost of drugs and antibiotics) and longer LOS in hospital and LOS in the ICU (Table 2).

**Figure 1:**
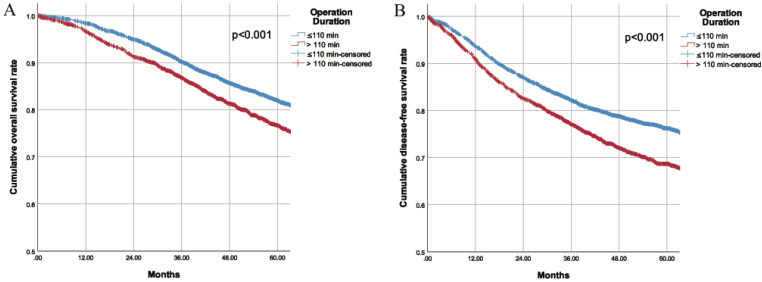
** A.** Overall survival of patients with different operation duration **B.** Disease free survival of patients with different operation duration

During a mean longitudinal follow-up period of 56.0 months (95% CI 55.2-56.9) after surgery, 1117 deaths occurred (17.9%) and 1515 patients (24.3%) had recurrences. The Kaplan-Meier cumulative survival curves for OS and DFS for patients in the two groups are shown in Fig. 1. The 5-year OS for patients was 82.0% in the SOD group and 76.7% in the LOD group (*P*<0.001). Similarly, the 5-year DFS was 76.2% in the SOD group and 68.7% in the LOD group (*P*<0.001). Furthermore, the OS and DFS were compared between the two matched cohorts after PSM. Likewise, obviously worse OS and DFS were observed in the LOD group in comparison with PSM controls (Supplementary Fig. 4). These results revealed that shorter operation duration was associated with higher OS and DFS in CRC patients undergoing colorectal resection. With each additional minute prolonging surgery, the risk of death increased by 0.4% and risk of recurrence/metastasis/death increased by 0.5%. Although the unit effect was quite small, the risk increased by 24% when the surgery was prolonged by one hour.

### Factors related to long-term prognosis

Univariate and multivariate Cox proportional hazards analyses were performed to evaluate the relationship between operation duration and long-term prognosis (OS and DFS) (Tables 3 and 4). On univariate COX regression, longer operation duration was found to be associated with poor prognosis, with a HR of 1.004 (1.002, 1.005) for OS and 1.004 (1.003, 1.005) for DFS. After adjusting for confounders such as sex, age, pTNM staging, tumor site, histological type, EMVI, PNI, CRM, blood haemoglobin and serum albumin level, number of lymph nodes examined, and surgical approach, operation duration was found to be an independent risk factor for OS with a HR of 1.004 (1.003, 1.005), and a HR of 1.005 (1.003, 1.006) for DFS. In addition to operation duration, gender, age, pTNM, tumor site, histological type, EMVI, PNI, CRM, hemoglobin, albumin, and number of lymph nodes extracted were all significantly associated with OS, and with DFS (P<0.001).

### Factors associated with operation duration

In order to explore the influencing factors of operation duration, we investigted the correlation between operation duration and other clinico-pathological characteristics. Operation methods, surgeons' qualifications, anesthesia methods, histological type, age, gender, BMI and tumor site were associated with operation duration (Table 5). More importantly, surgical approach and surgeons' qualifications had a meaningful correlation coefficient with r=0.464 and 0.450 respectively, which indicated that open surgery and surgery by junior surgeons had longer operation duration.

## Discussion

Our study suggests that longer operation duration is an important independent risk factor for prognosis of patients undergoing colorectal surgery, and increases total costs in hospital, LOS in hospital, LOS in the ICU, and costs associated with of drugs and antibiotics. Concerning long-term prognosis of patients, prolonged operation duration was associated with poorer OS and DFS after surgery. Furthermore, our results revealed that surgical approach and surgeons' qualifications are important factors affecting operation duration, even when the analysis was restricted to surgeons with more than 10 years of surgical experience.

Our results are consistent with previous studies showing that prolonged operation duration was associated with in-hospital costs and the prognosis of patients. Unsurprisingly, prolonged operation duration increased operative field exposure time, which might increase bleeding and stress response. It is well known that systemic inflammatory response can increase LOS and affect the immune system. Importantly, the current study indicated that surgeons' qualifications had a significant effect on operation duration. Therefore, it may be possible to reduce the operation time and thus improve the prognosis of patients by improving the surgical skills of surgeons and strengthening the cooperation of all parties. In addition, surgical approach is also an important factor affecting the operation duration. During the period 2011-2016, laparoscopic colorectal surgery had only recently been introduced at our hospital. Influenced by the learning curve, the duration of endoscopic surgery was significantly longer than that of open surgery. In recent years, with improvements in the level of endoscopic surgery, the duration of endoscopic surgery has decreased significantly, so the advantage of endoscopic surgery is gradually emerging. At present, there is little difference in duration between endoscopic surgery and open surgery. Therefore, the key to reducing operation duration is to improve the surgical skills of the surgeon and strengthen team cooperation.

Previous studies have suggested that prolonged operation duration increases mortality 14, 15, infection risk 7, 13, LOS 10, and the cost of operations 16. Consistent with these studies, our results also suggest that longer operation duration is related to poor prognosis, including increased costs, increased LOS in hospital and in the ICU, and lower OS and DFS. Additionally, Hernandez et al. found that surgical duration above 120 minutes was associated with increased risk of proximal deep vein thrombosis 17. However, Wills et al. studied 103044 patients undergoing total hip arthroplasty (THA) from National Surgical Quality Improvement Program (NSQIP) data, and their results agreed with the relationship of prolonged operation duration and superficial surgical site infection, but not deep surgical site infection, organ/space infections, or deep vein thrombosis 18. Till now, there are only two retrospective studies investigated the impact of prolonged operative time on early postoperative morbidity after colorectal cancer surgery and the conclusions are contradictory. The first study suggested that longer operating times may increase postoperative complication rates for CRC patients with higher BMI 19. The second study found that there was no relationship between prolonged operation duration and postoperative complications morbidity of patients with rectal cancer undergoing open or robotic surgical resection 9. With these, we assessed in a large-scale cohort of 6224 patients who underwent radical surgery for colorectal cancer. Our study revealed that prolonged operation duration was not only associated with short-term prognosis but also the long-term prognosis (5-year OS and DFS) of patients.

Most importantly, we pointed out the key factors that affected operation duration. In our research, although some factors were statistically associated with operation duration, only surgical approach and surgeons' qualification are meaningful parameters that can be improved. While surgeon experience would affect operation duration, the team familiarity and collaborations could reduce operation duration by a mean of 59 and 22 minutes, respectively 20. Besides our study, others have suggested that age, gender, tumor site, and anesthesia methods 21 were related to operation duration. Further studies are needed to identify predictive factors for operation duration to guide surgeons in their choice of procedures. It is interesting that several meta-analyses have suggested that robotic operation had longer operation duration than open, laparoscopic, and transanal mesorectal excision 22, 23, but with a shorter hospital stay. So, the mechanism of operation duration on prognosis is complex and warrants further study.

Although the exact mechanisms underlying the positive association between prolonged operation duration and clinical outcomes for CRC patients are not fully understood, plausible links do exist. Possible explanations for the impact of prolonged operation duration on outcomes entail greater surgical trauma, increased risk of venous thromboembolism, augmented exposure to air pathogens, increased suppression of immune function, and decreased nutrient absorption. It is conceivable that longer operative times may also reflect more complex surgical procedures, a wider range of tumors, greater surgical trauma, increased surgical team fatigue, and more technical errors 24, 25. It is well established that prolonged operation duration can lead to the simultaneous presence of blood stasis, increased coagulation, and endothelial damage, contributing to the risk of venous thromboembolism 25. Theoretically, longer operative time predisposes the patient to increased exposure to microbes and decreased prophylactic effect of antibiotics, thus leading to subsequent surgical site infections 26. Finally, it has been shown that longer operative time may result in more delayed postoperative gastrointestinal function recovery, postoperative intestinal obstruction, and decreased absorption of nutrients 27. Each of these different types of complications could be expected to result in worse postoperative outcomes.

Our research has several limitations. First of all, as a retrospective observational study, the time span of enrollment is wide, and the records of postoperative complications in the medical history of patients are not complete, making it difficult to obtain complete data on postoperative complications which is a good indicator for short-term prognosis. Additionally, there are many factors that affect the five-year OS and DFS of CRC patients other than the collected variables. The results of the study may be confounded by these uncollected factors. Thus, our conclusions need to be verified by more prospective clinical studies.

## Conclusions

It was found that long operation duration was associated with poor short-term prognosis and long-term mortality. Enhancing surgeons' operative abilities may have a beneficial effect for improving the prognosis of patients. Further studies are needed to confirm the impact of operation duration on patients' prognosis and to explore the mechanisms of the relationship.

## Supplementary Material

Supplementary figures and table.

## Figures and Tables

**Table 1:** Characteristics of patients with different operation duration

		≤110 min		>110 min				
Characteristics		N (%)	Mean (±SD)	N (%)	Mean (±SD)	Chi2	t	P value
Gender	Male	2127(57.99)		1600(62.60)		13.32		<0.001*
	Female	1541(42.01)		956(37.40)				
Age Group (year)	<45	399(10.88)		334(13.07)		18.47		0.001*
	45-54	685(18.68)		510(19.95)				
	55-64	1271(34.65)		900(35.21)				
	65-74	903(24.62)		591(23.12)				
	75+	410(11.18)		221(8.65)				
Surgical Approach	Open Surgery	3488(95.09)		1577(61.70)		1108.54		<0.001*
	Laparoscope	180(4.91)		979(38.30)				
BMI kg/m^2^	Normal (18.5-23.9)	2096(61.29)		1324(38.71)		30.488		<0.001*
	Underweight (<18.5)	236(63.96)		133(36.04)				
	Overweight (24-29.9)	1264(55.37)		1019(44.63)				
	Obesity (≥30)	70(48.28)		75(51.72)				
pTNM Staging	I	710(19.36)		515(20.15)		0.65		0.722
	II	1309(35.69)		910(35.60)				
	III	1649(44.96)		1131(44.25)				
Tumor Site	Rectum	1997(55.49)		1560(61.47)		37.48		<0.001*
	Left Colon	866(24.06)		449(17.69)				
	Right Colon	736(20.45)		529(20.84)				
Histological type	Adenocarcinoma	3241(88.36)		2229(87.21)		7.97		0.019*
	Mucinous adenocarcinoma	377(10.28)		268(10.49)				
	Signet-ring cell carcinoma	50(1.36)		59(2.31)				
EMVI	-	2885(78.65)		2039(79.77)		1.14		0.285
	+	783(21.35)		517(20.23)				
PNI	-	2930(79.88)		2056(80.44)		0.29		0.587
	+	738(20.12)		500(19.56)				
CRM	-	3643(99.32)		2519(98.55)		8.96		0.003*
	+	25(0.68)		37(1.45)				
Anesthesia	GA+EA	2248(61.29)		1477(57.79)		7.68		0.006*
	GA	1420(38.71)		1079(42.21)				
ASA	I	1249(34.05)		871(34.08)		7.11		0.029*
	II	2335(63.66)		1598(62.52)				
	III-V	84(2.29)		87(3.40)				
Number of lymph nodes examined			16.69±6.42		16.64±7.24		0.281	0.779
Hemoglobin (g/l)			126.76±20.30		125.95±21.01		1.502	0.133
Number of platelets (10^9/L)			230.71±78.50		232.41±80.27		-0.821	0.412
Albumin (g/l)			42.05±3.79		42.03±4.00		0.163	0.871
Creatinine (μmol/l)			70.84±17.84		71.41±18.54		-1.185	0.236
Alkaline phosphatase (IU/L)			73.72±21.92		75.23±28.39		-2.056	0.040*
Total cost (RMB)			43559±13298		48769±17031		-13.531	<0.001*
Length of stay in hospital (days)			14.91±6.64		16.40±7.41		-8.341	<0.001*
Length of stay in ICU (days)			0.18±1.17		0.24±1.22		-1.990	0.047*
Cost of drug (RMB)			15530±8130		17270±9779		-7.637	<0.001*
Cost of antibiotics (RMB)			940±1158		1080±1377		-4.362	<0.001*
Total		3668(58.93)		2556(41.07)				

**Table 2:** Univariate regression of operation duration on patients' short-term effect

	β	t	P value
	Total Fees (RMB)		
Constant	36323.520	67.800	<0.001*
operation duration	84.463	18.674	<0.001*
	Inpatient days (days)		
Constant	12.806	50.879	<0.001*
operation duration	0.024	11.509	<0.001*
	Drug Fees (RMB)		
Constant	12704.484	39.820	<0.001*
operation duration	31.892	11.840	<0.001*
	Antibiotic fees (RMB)		
Constant	708.937	15.620	<0.001*
operation duration	2.600	6.785	<0.001*

**Table 3:** Univariate and multivariate regression of factors affecting OS using Cox regression

Characteristics	Univariate			Multivariate		
	β	P value	HR (95% CI)	β	P value	HR (95% CI)
Female/Male	-0.103	0.095	0.902(0.800,1.018)	-0.213	0.002*	0.808(0.706,0.924)
Age Group		<0.001*			<0.001*	
45-54/<45	-0.003	0.979	0.997(0.785,1.266)	0.213	0.103	1.237(0.958,1.598)
55-64/<45	0.039	0.727	1.039(0.837,1.290)	0.288	0.016*	1.334(1.055,1.686)
65-74/<45	0.274	0.015*	1.315(1.054,1.641)	0.395	0.002*	1.484(1.162,1.896)
75+/<45	0.959	<0.001*	2.610(2.074,3.283)	1.076	<0.001*	2.932(2.265,3.795)
Laparoscope/Open Surgery	0.002	0.977	1.002(0.854,1.176)			
BMI		0.007*				
Underweight/Normal	0.300	0.010*	1.349(1.075,1.693)			
Overweight/Normal	-0.058	0.366	0.943(0.831,1.070)			
Obesity/Normal	-0.403	0.084	0.668(0.423,1.055)			
pTNM		<0.001*			<0.001*	
II/I	0.528	<0.001*	1.695(1.343,2.140)	0.420	0.001*	1.522(1.189,1.947)
III/I	1.368	<0.001*	3.927(3.171,4.862)	1.030	<0.001*	2.801(2.217,3.538)
Site		<0.001*			0.007*	
Left Colon/Rectum	-0.159	0.001*	0.853(0.730,0.997)	-0.257	0.002*	0.773(0.656,0.911)
Right Colon/Rectum	-0.010	0.002*	0.990(0.853,1.149)	-0.137	0.121	0.872(0.734,1.037)
histological type		<0.001*			<0.001*	
mucinous adenocarcinoma /adinocacinoma	0.299	0.001*	1.349(1.131,1.609)	0.281	0.003*	1.325(1.098,1.598)
Signet-ring cell carcinoma /adinocacinoma	1.138	<0.001*	3.121(2.314,4.211)	0.615	<0.001*	1.850(1.326,2.583)
EMVI+/-	0.968	<0.001*	2.634(2.331,2.975)	0.573	<0.001*	1.773(1.539,2.043)
PNI+/-	0.860	<0.001*	2.363(2.086,2.677)	0.479	<0.001*	1.615(1.405,1.856)
CRM+/-	1.557	<0.001*	4.747(3.389,6.648)	1.180	<0.001*	3.254(2.281,4.641)
GA+EA/ GA	-0.020	0.755	0.980(0.866,1.110)			
ASA		<0.001*				
II/I	0.212	0.001*	1.236(1.086,1.406)			
III-V/I	0.728	<0.001*	2.071(1.517,2.828)			
Haemoglobin(g/l)	-0.009	<0.001*	0.991(0.988,0.993)	-0.008	<0.001*	0.992(0.988,0.996)
Albumin (g/l)	-0.070	<0.001*	0.933(0.919,0.947)	-0.042	<0.001*	0.959(0.941,0.976)
Operation duration	0.004	<0.001*	1.004(1.002,1.005)	0.004	<0.001*	1.004(1.003,1.005)
Number of lymph nodes examined	-0.030	<0.001*	0.971(0.961,0.980)	-0.037	<0.001*	0.964(0.953,0.974)

**Table 4:** Univariate and multivariate regression of factors affecting DFS using Cox regression

Characteristics	Univariate			Multivariate		
	β	P value	HR (95% CI)	β	P value	HR (95% CI)
Female/Male	0.002	0.977	0.918(0.828,1.019)	-0.173	0.003*	0.841(0.749,0.945)
Age Group		<0.001*			<0.001*	
45-54/<45	-0.035	0.726	0.966(0.796,1.173)	0.148	0.163	1.159(0.942,1.427)
55-64/<45	-0.021	0.813	0.979(0.821,1.167)	0.199	0.041*	1.220(1.008,1.476)
65-74/<45	0.071	0.448	1.074(0.894,1.290)	0.209	0.043*	1.232(1.007,1.508)
75+/<45	0.558	<0.001*	1.748(1.435,2.129)	0.686	<0.001*	1.985(1.590,2.478)
Laparoscope/ Open Surgery	0.002	0.977	1.002(0.877,1.145)	-0.165	0.042*	0.848(0.724,0.994)
BMI		0.011*				
Underweight/Normal	0.191	0.063	1.210(0.990,1.480)			
Overweight/Normal	-0.111	0.045*	0.895(0.803,0.998)			
Obesity/Normal	-0.262	0.158	0.769(0.535,1.107)			
pTNM		<0.001*			<0.001*	
II/I	0.458	<0.001*	1.582(1.310,1.909)	0.411	<0.001*	1.509(1.235,1.843)
III/I	1.205	<0.001*	3.336(2.805,3.966)	0.904	<0.001*	2.470(2.040,2.990)
Site		0.018*			0.005*	
Left Colon/Rectum	-0.195	0.005*	0.823(0.719,0.942)	-0.236	0.001*	0.790(0.685,0.911)
Right Colon/Rectum	-0.039	0.550	0.961(0.845,1.093)	-0.098	0.199	0.906(0.780,1.053)
histological type		<0.001*			0.011*	
mucinous adenocarcinoma /adinocacinoma	0.199	0.013*	1.220(1.044,1.426)	0.175142	0.039*	1.191(1.009,1.407)
Signet-ring cell carcinoma /adinocacinoma	0.917	<0.001*	2.501(1.905,3.284)	0.356917	0.020*	1.429(1.059,1.929)
EMVI+/-	0.871	<0.001*	2.389(2.149,2.657)	0.496	<0.001*	0.000(0.000,0.000)
PNI+/-	0.810	<0.001*	2.249(2.019,2.505)	0.465	<0.001*	1.191(1.009,1.407)
CRM+/-	1.460	<0.001*	4.305(3.177,5.832)	0.993	<0.001*	1.429(1.059,1.929)
Epidural anesthesia/ General anesthesia	-0.020	0.708	0.980(0.883,1.088)			
ASA		0.002*				
II/I	0.143	0.011*	1.153(1.034,1.287)			
III-V/I	0.448	0.002*	1.565(1.178,2.079)			
Haemoglobin (g/l)	-0.007	<0.001*	0.993(0.991,0.995)	-0.007	<0.001*	0.993(0.990,0.997)
Albumin (g/l)	-0.041	<0.001*	0.960(0.948,0.973)	-0.021	0.008	0.979(0.964,0.995)
Operation duration	0.004	<0.001*	1.004(1.003,1.005)	0.005	<0.001*	1.005(1.003,1.006)
Number of lymph nodes examined	-0.037	<0.001*	0.964(0.953,0.974)	-0.031	<0.001*	0.970(0.961,0.979)

**Table 5:** Correlation between characteristics of surgery and duration of surgery

Characteristics	Correlation Coefficient	P value
Operation methods: laparoscope/open surgery	0.464	<0.001*
Surgeon's qualification: Young/Old	0.450	<0.001*
Anesthesia methods: Epidural/ General anesthesia	0.028	0.029*
Histological type	0.025	0.048*
ASA	-0.003	0.789
Staging	-0.017	0.180
Number of lymph nodes examined	-0.024	0.056
Age	-0.044	0.001*
Female/male	-0.059	<0.001*
BMI	0.074	<0.001*
Tumor site	-0.063	<0.001*
