# Supplementary material for: Impact of operation duration on short-term and long-term prognosis in patients undergoing radical colorectal surgery
Source: J Cancer. 2022 Jan 16;13(4):1160–7. doi: 10.7150/jca.65817 (PMC8899385; doi:10.7150/jca.65817)
Supplement: Supplementary file 1 — Supplementary figures and table. [file jcav13p1160s1.pdf]

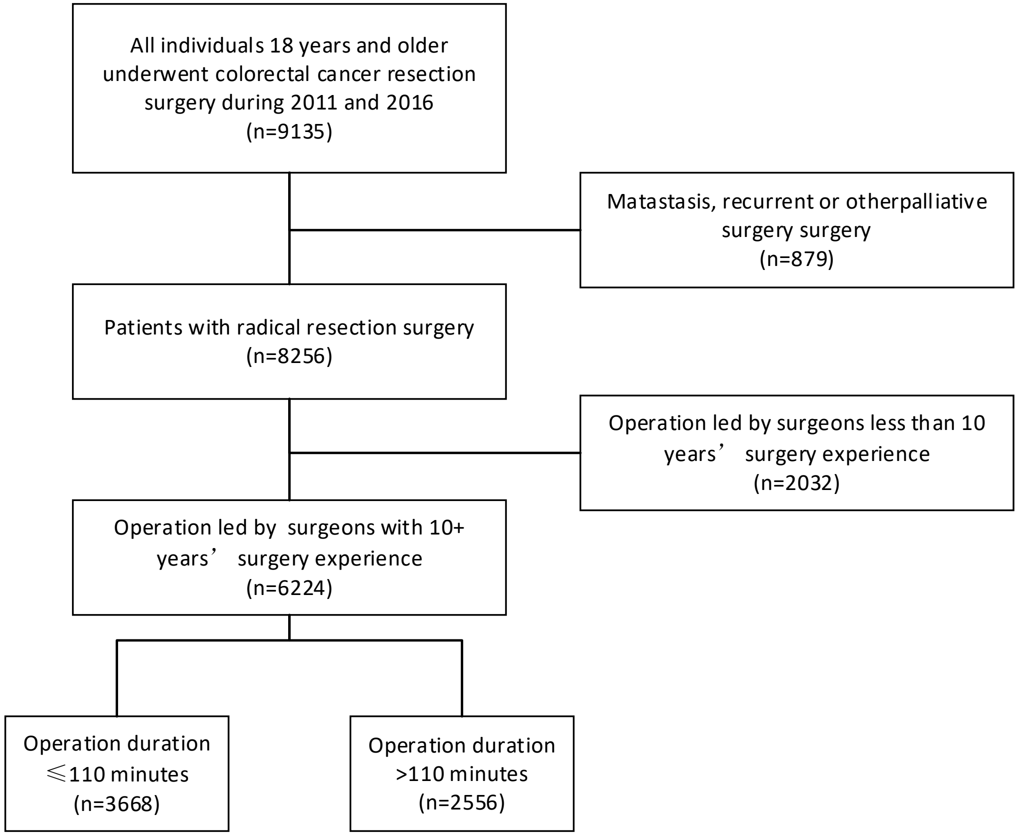

Supplementary Figure 1 Diagram of patients’ inclusion in the study

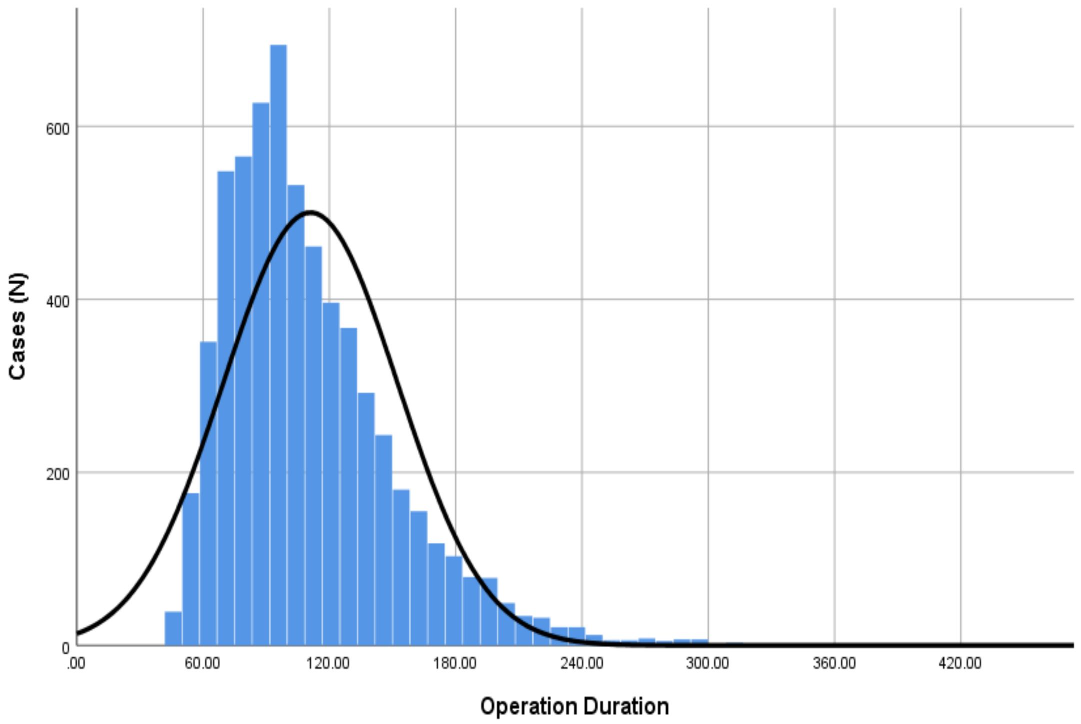

Supplementary Figure 2 Distribution of CRC operation duration

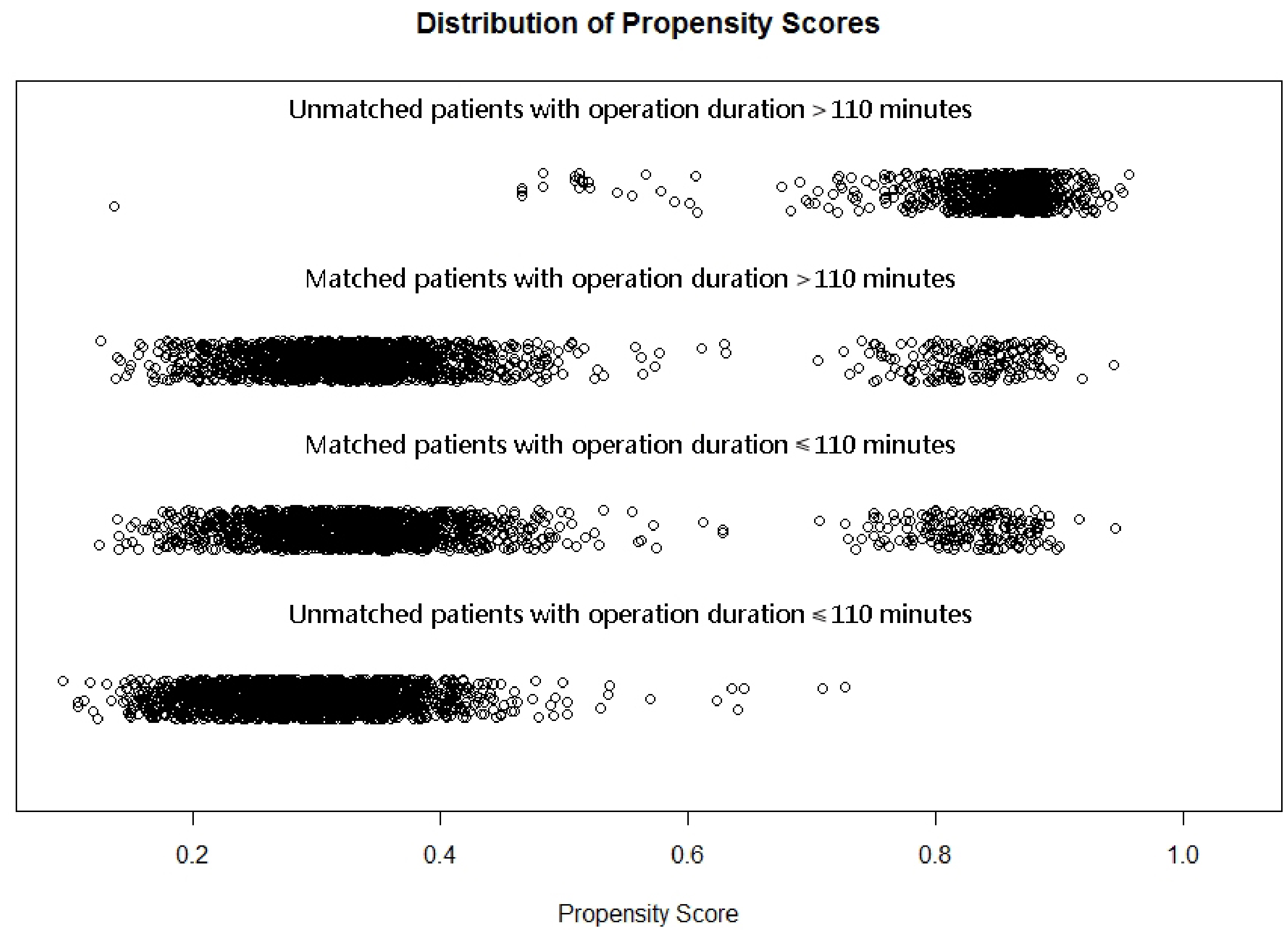
Distribution of Propensity Scores
Unmatched patients with operation duration > 110 minutes
Matched patients with operation duration > 110 minutes
Matched patients with operation duration ≤ 110 minutes
Unmatched patients with operation duration ≤ 110 minutes
0.2
0.4
0.6
0.8
1.0
Propensity Score

Supplementary Figure 3 Distribution of propensity matching: operation duration > 110minutes matching operation duration≤110minutes.

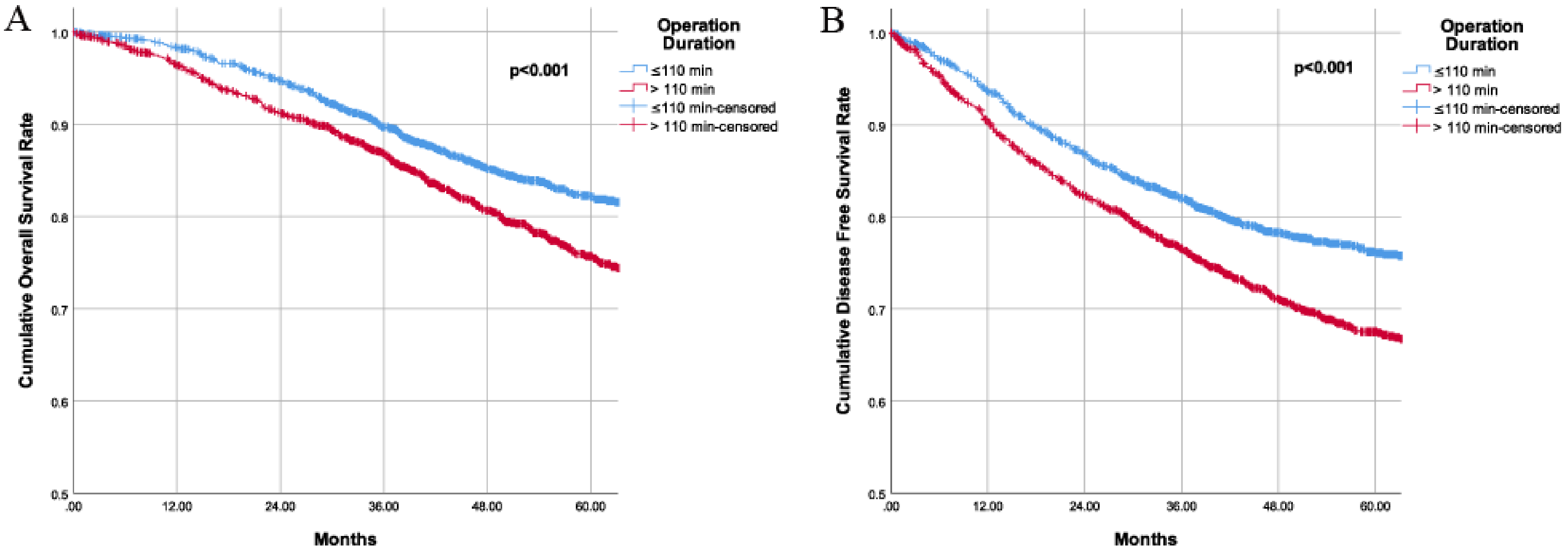

Supplementary Figure 4 A. Overall survival of patients with different operation duration after PSM

B. Disease free survival of patients with different operation duration after PSM

Supplementary Table 1 Baseline characteristics after PSM – 1:1 matching of patients with different operation duration

		SOD (≤110 min)		LOD (>110 min)		P value	SMD
Characteristics		N (%)	Mean (±SD)	N (%)	Mean (±SD)		
Gender	Male	1070(49.33)		1099(50.67)		0.325	0.035
	Female	660(51.12)		631(48.88)			
Age Group (year)	＜45	211(49.88)		212(50.12)		0.768	0.046
	45-54	344(51.96)		318(48.04)			
	55-64	616(49.96)		617(50.04)			
	65-74	406(49.45)		415(50.55)			
	75+	153(47.66)		168(52.34)			
Surgical Approach	Open Surgery	1553(50.00)		1553(50.00)			
	Laparoscope	177(50.00)		177(50.00)			
pTNM Staging	I	334(50.15)		332(49.85)		0.848	0.019

	II	631(50.56)	617(49.44)		
	III	765(49.48)	781(50.52)		
Tumor Site	Rectum	1005(49.95)	1007(50.05)	0.89	0.027
	Left Colon	307(48.96)	320(51.04)		
	Right Colon	402(51.02)	386(48.98)		
Histological type	Adenocarcinoma	1507(50.12)	1500(49.88)	0.867	0.018
	Mucinous adenocarcinoma	189(48.84)	198(51.16)		
	Signet-ring cell carcinoma	34(51.52)	32(48.48)		
EMVI	-	1382(50.07)	1378(49.93)	0.899	0.006
	+	348(49.71)	352(50.29)		
PNI	-	1392(50.00)	1392(50.00)	1	<0.001
	+	338(50.00)	338(50.00)		
CRM	-	1716(50.09)	1710(49.91)	0.389	0.035
	+	14(41.18)	20(58.82)		

Number of lymph nodes examined		125.91(21.50)	125.73(20.95)	0.738	0.011
Hemoglobin (g/l）		41.86(3.84)	41.82(3.98)	0.8	0.009
Albumin (g/l)		41.86(3.84)	41.82(3.98)	0.786	0.009
Total	1730(100)		1730(100)	-	-
